# Riluzole: a therapeutic strategy in Alzheimer’s disease by targeting the WNT/β-catenin pathway

**DOI:** 10.18632/aging.102830

**Published:** 2020-02-08

**Authors:** Alexandre Vallée, Jean-Noël Vallée, Rémy Guillevin, Yves Lecarpentier

**Affiliations:** 1 DACTIM-MIS, Laboratory of Mathematics and Applications (LMA), University of Poitiers, CHU de Poitiers, Poitiers, France; 2 CHU Amiens Picardie, University of Picardie Jules Verne (UPJV), Amiens, France; 3 Laboratory of Mathematics and Applications (LMA), University of Poitiers, Poitiers, France; 4 Centre de Recherche Clinique, Grand Hôpital de l’Est Francilien (GHEF), Meaux, France

**Keywords:** Riluzole, Alzheimer's disease, WNT pathway, glutamate, oxidative stress

## Abstract

Alzheimer’s disease (AD) is a neurodegenerative disease, where the etiology remains unclear. AD is characterized by amyloid-(Aβ) protein aggregation and neurofibrillary plaques deposits. Oxidative stress and chronic inflammation have been suggested as causes of AD. Glutamatergic pathway dysregulation is also mainly associated with AD process. In AD, the canonical WNT/β-catenin pathway is downregulated. Downregulation of WNT/β-catenin, by activation of GSK-3β-induced Aβ, and inactivation of PI3K/Akt pathway involve oxidative stress in AD. The downregulation of the WNT/β-catenin pathway decreases the activity of EAAT2, the glutamate receptors, and leads to neuronal death. In AD, oxidative stress, neuroinflammation and glutamatergic pathway operate in a vicious circle driven by the dysregulation of the WNT/β-catenin pathway. Riluzole is a glutamate modulator and used as treatment in amyotrophic lateral sclerosis. Recent findings have highlighted its use in AD and its potential increase power on the WNT pathway. Nevertheless, the mechanism by which Riluzole can operate in AD remains unclear and should be better determine. The focus of our review is to highlight the potential action of Riluzole in AD by targeting the canonical WNT/β-catenin pathway to modulate glutamatergic pathway, oxidative stress and neuroinflammation

## INTRODUCTION

Alzheimer’s disease (AD) is one of the major neurodegenerative disease, but its etiology remains unclear. AD is marked by two major postmortem hallmarks; amyloid-(Aβ) protein aggregation formed by plaque deposits and tau protein hyperphosphorylation which results in neurofibrillary tangles. In AD, the common symptoms are cognitive function dysregulation, memory loss and neurobehavioral manifestations [1]. Other cognitive and behavioral symptoms are poor facial recognition ability, social withdrawal, increase in motor agitation and wandering likelihood [2, 3]. Aging is the main risk factors of AD [4]. Affected neural circuits in aging and AD are the same, and involving glutamatergic pathway, oxidative stress and neuroinflammation [5, 6]. Glutamatergic neurons are vulnerable to damages in AD and in aging [7–9]. Oxidative stress and neuroinflammation are considered as mainly underlying causes of AD [10, 11]. Increase of oxidative stress can be an early indication of AD [12, 13]. In AD, the accumulation of Aβ protein leads to the decrease of the WNT/β-catenin pathway [14]. Diminution of β-catenin decreases phosphatidylinositol 3-kinase (PI3K)-protein kinase B (Akt) (PI3K/Akt) pathway activity [15, 16]. Inhibition of WNT/β-catenin/PI3K/Akt pathway enhances oxidative stress in mitochondria of AD cells [17]. Thus, activation of the WNT/β-catenin pathway may be an interesting therapeutic target for AD [18, 19].

Riluzole is a glutamate modulator and used as treatment in amyotrophic lateral sclerosis [20]. Moreover, use of Riluzole is associated with prevention of age-related cognitive decline [21]. Riluzole administration can be correlated with induction of dendritic spines clustering [21] depending on glutamatergic neuronal activity [22, 23]. In mutant mouse and rat model of AD, Riluzole can prevent age-related cognitive decline [21, 24]. Moreover, Riluzole is associated with the rescue age-related gene expression changes in hippocampus of rats [6]. Hippocampus region is responsible for learning and memory and is one of the regions compromised by AD progression [25, 26].

Nevertheless, the mechanism by which Riluzole can operate in AD remains unclear and should be better determine. The focus of our review is to highlight the potential action of Riluzole in AD by targeting the canonical WNT/β-catenin pathway to modulate glutamatergic pathway, oxidative stress and neuroinflammation.

## HALLMARKS OF AD: OXIDATIVE STRESS AND NEUROINFLAMMATION

AD manifestations are characterized by senile plaques, due to the extracellular accumulation of the amyloid β (Aβ) protein [27], and neurofibrillary tangles (NFTs), caused by hyperphosphorylated tau aggregation [28].

Aβ is produced by the sequential cleavage of the Amyloid Precursor Protein (APP), controlled by the β-secretase (BACE-1) and complex of gamma-secretase [29]. NFTs is formed by the aggregation of hyperphosphorylated microtubule-associated protein (MAP) tau. Tau is a microtubule-stabilizing protein maintaining the structure of neuronal cells and the axonal transport. In AD, multiple kinases phosphorylate Tau in an aberrantly manner. These kinases are the Glycogen synthase kinase-3β (GSK-3β), the cyclin-dependent protein kinase-5 (CDK5), the Dual specificity tyrosine-phosphorylation-regulated kinase 1A (DYRK1A), the Calmodulin-dependent protein kinase II (CAMKII), and the Mitogen-activated protein kinases (MAPKs) are the best known [30–32].

Some pathways including genetic factors, neuroinflammation correlated with neurotoxicity, oxidative stress and cytokine release, are considered as possible underlying causes [10, 11]. Aβ and NFTs involve neuroinflammation and oxidative damages resulting in progressive neuronal degeneration. Oxidative stress enhancement can be an indication of AD [13].

In AD, mitochondrial damages enhance the production of ROS (reactive oxygen species) but diminish the production of ATP [33]. Mitochondrial damages affect cell function by enhancing the release of ROS leading to cell damage and death. Energy depletion is caused by the disruption of oxidative phosphorylation [34]. Thus, both the dysregulation of mitochondrial activity and oxidative stress enhancement are responsible to dementia and neuronal cell death [35–37].

Numerous cellular pathways are altered by Aβ-induced oxidative stress [38]. Neurotoxic effects are induced by Aβ peptide through the enhancement of oxidative stress and damages on the membrane, mitochondrial function and lipids production [39]. NADPH dehydrogenase (complex I) generates superoxide from oxidative phosphorylation into the mitochondrial respiratory chain [40]. Complex I and complex IV (cytochrome c oxidase) deficiencies are initiated by Aβ. These deficiencies lead to ROS generation [41]. Mitochondrial-derived ROS correlated with Aβ, are inhibited in resistant relative to sensitive cells. Through the diminution of the mitochondrial respiration chain, Aβ-resistant cells are less likely to generate ROS and are mainly resistant to depolarization of the mitochondria [17].

Amyloid oligomers complex into the lipid bilayer and lead to the peroxidation of lipids, proteins and biomolecule damages [42]. Membrane alteration generated by the accumulation of Aβ are induced by the influx of Ca^2+^. This leads to the alteration of the homeostasis of Ca^2+^ leading to mitochondrial dysregulation and neuronal death. Diminution of the activity of Glutathione (GSH) is responsible for the increase of Ca^2+^ release and ROS accumulation [43]. Then, ROS accumulation affects DNA transcription, DNA oxidation and the activity of the target proteins [44, 45]. Tau leads to the dysregulation of the mitochondrial activity, which dysregulates energy production, enhances ROS and nitrogen species (RNS) production [46]. ROS and RNS alters the integrity of cell membranes to induce failure of synapses [47]. ROS production activates pro-inflammatory gene transcription and cytokines release, including interleukin-1 (IL-1) and tumor necrosis factor-α (TNF-α), responsible for neuroinflammation [37]. Aβ-related inflammatory compound of the disease is one of the main targets to control AD development [48]. Aβ stimulates inflammation leading to damage and neuronal death [49].

Numerous studies have shown the link between neuroinflammation and oxidative stress [50]. NF-κB induces the production of ROS and RNS leading to neuronal damages [51, 52]. NF-κB activates COXX-2 and cytosolic phospholipase A2 which stimulate prostaglandins production leading to oxidative stress [53]. Production of peroxide, through the involvement of iNOS and NF-κB pathway, is associated with dysregulation of the glucose metabolism [54]. IL-1 can stimulate GSH production in astrocytes through a NF-κB dependent pathway [55].

## GLUTAMATERGIC PATHWAY IN AD

Glutamate is a key excitatory neurotransmitter in the CNS, responsible for fast excitatory neurotransmission. In neurons, glutamate is stored in synaptic vesicles, from where it is released. The release of glutamate leads to an increase in glutamate concentration in the synaptic cleft, which binds the ionotropic glutamate receptors. Glutamate is removed from the synaptic cleft and transported to astrocytes by glutamate transporters (such as GLT-1 or excitatory amino acid transporters 1 and 2: EAATs 1 and 2) to prevent overstimulation of the glutamate receptor [56]. Astrocytes clear >90% of excess glutamate by EAATs and play a major role in the glutamate/glutamine cycle. Following glutamate uptake, glutamine synthetase (GS) catalyzes the ATP-dependent reaction of glutamate and ammonia into glutamine. Glutamine is released and in turn is taken up by neurons for conversion back to glutamate by glutaminase.

In a physiological state, in astrocytes, β-catenin activates the gene expression of EAAT2 and GS [57]. This allows the re-uptake of glutamate from the synaptic cleft by astrocytes through EAAT2. Glutamate is then metabolized by GS.

In AD, EAAT2 expression is decreased [58]. The over-accumulation of glutamate in the synaptic cleft leads to excitotoxicity that impairs glutamate receptors located on the post-synaptic side of the cleft. This phenomenon leads to calcium overload, mitochondrial dysfunction, apoptosis and ultimately death of the post-synaptic neuron. Cell death is restricted to post-synaptic neurons. The decrease if glutamate transmission is significantly associated with neuronal death and loss of synapse [56]. Moreover, the downregulation of glutamate transport is correlated with the decrease of EAAT2 expression in AD [58].

Some animal models of AD have shown the importance of NMDA receptors (glutamatergic N-methyl-D-aspartate) in AD and the affection of glutamatergic synapses [59, 60].

Synaptic dysregulation is one the main mechanism involved in AD [28] which is present at early step of AD development [61]. Moreover, Aβ expression is closely associated with glutamatergic pathway expression [62]. Excessive activation of extra-synaptic NMDA receptors [63]and excessive downregulation of synaptic NMDA receptors [64] lead to increase of Aβ release [65].

## OXIDATIVE STRESS, NEUROINFLAMMATION AND GLUTAMATERGIC PATHWAY IN AD

Oxidative stress leads to the loss of cell homeostasis by mitochondrial oxidants overproduction [66]. The development of oxidative stress in AD compromises astrocyte function leading to impairment of glutamate transport and then increasing excitotoxicity to neurons [67]. Aβ interaction on the membrane of astrocytes induces calcium changes. Mitochondrial dysregulation in astrocytes is associated with a mitochondrial depolarization, increased conductance and membrane permeability [68]. The formation of calcium selective channels on membrane could be induced by Aβ into astrocytes generating a change in the conductance [69]. Aβ insertion in membrane changes the structure of membrane [70]. In AD, astrocytes appear as the primary target of Aβ, and oxidative stress enhancement is associated with the alteration of calcium intracellular signaling [69]. Astrocytes have a major role in neuronal integrity. Changes in cytokines and oxidative damages in astrocytes increase neurotoxicity and vulnerability of neurons [67]. In parallel a vicious and positive crosstalk is observed between oxidative stress and neuroinflammation. NF-κB activation induces the generation of prostaglandins and oxidative stress [53] whereas oxidative stress can stimulate in a direct feedback NF-κB pathway [50]. Thus, interesting drugs should consider the modulation of astrocyte activity to reduce both inflammation and oxidative stress.

## THE CANONICAL WNT/β-CATENIN PATHWAY (FIGURE 1)

The Wingless/Int (WNT) pathway is a family of secreted lipid-modified glycoproteins [71]. Several signaling are mediated by this pathway, including fibrosis and angiogenesis [72–74].

**Figure 1:**
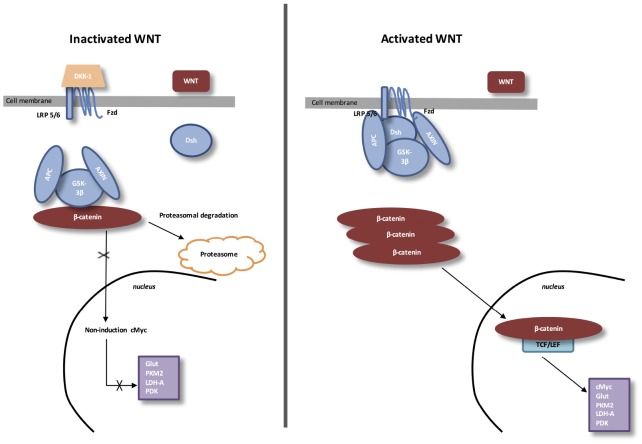
**The canonical WNT/β-catenin pathway.** Inactivated WNT: Under physiologic circumstances, the cytoplasmic β-catenin is linked to its destruction complex, consisting of APC, AXIN and GSK-3β. β-catenin is phosphorylated by GSK-3β. Thus, phosphorylated β-catenin is destroyed into the proteasome. Then, cytoplasmic level of β-catenin is kept low in the non-presence of WNT ligands. If β-catenin is not accumulated in the nucleus, the TCF/LEF complex does not stimulate the target genes. DKK1 inhibits the WNT/β-catenin pathway through the bind to WNT ligands or LRP5/6. Activated WNT: When WNT ligands activate both FZD and LRP5/6, DSH is stimulated and phosphorylated by FZD. Phosphorylated DSH in turn activates AXIN, which comes off β-catenin destruction complex. Thus, β-catenin escapes from phosphorylation and then accumulates in the cytoplasm. The accumulated cytosolic β-catenin moves into the nucleus, where it interacts with TCF/LEF and stimulates the transcription of target genes.

During eye development, WNT/β-catenin pathway activity is highly mediated. Then, a dysfunction of the WNT/β-catenin pathway leads to several ocular malformations due to defects in cell fate differentiation and determination [75]. During the development of lens, the WNT/β-catenin pathway is stimulated in the periocular surface ectoderm and lens epithelium [76, 77]. For the retinal development, the WNT/β-catenin pathway is stimulated in the dorsal optic vesicle and then, participates to the activation of RPE at the optic vesicle step. At this level, WNT/β-catenin pathway is contained to the peripheral RPE [78]. The retinal vascular initiation is mainly modulated by the expression of the WNT/β-catenin pathway [75]. In the retinal vascular system, WNT/β-catenin pathway is controlled by the erythroblast transformation-specific (ETS) transcription factor Erg. Erg has a major and key role in angiogenesis [79]. Erg modulates the WNT/β-catenin pathway by promoting β-catenin stability and by regulating the transcription of Frizzled 4 (FZD4) [79].

Stimulation of FZD4/β-catenin signaling needs the presence of the complex LRP5 /LRP6 [80]. LRP5 has a main role while LRP6 presents a minor role in the retinal vascularization [81, 82]. Disheveled (Dsh) forms a complex with Axin, and this prevents the phosphorylation of β-catenin by glycogen synthase kinase-3β (GSK-3β). Then, β-catenin accumulation in the cytosol is observed and translocates to the nucleus to bind T-cell factor/lymphoid enhancer factor (TCF/LEF) co-transcription factors. This nuclear bind allows the transcription of WNT-responsive genes, such as cyclin D1, c-Myc, PDK1, MCT-1 [83, 84].

WNT ligands absence is associated with cytosolic β-catenin phosphorylation by GSK-3β.

A destruction complex is composed by tumor suppressor adenomatous polyposis coli (APC), Axin, GSK-3β and β-catenin. Then, phosphorylated β-catenin is destroyed in the proteasome. WNT inhibitors, including DKKs and SFRPs, control the WNT/β-catenin pathway by preventing its ligand-receptor interactions [85]. GSK-3β, a neuron-specific intracellular serine-threonine kinase, is the major inhibitor of the WNT pathway [86]. GSK-3β regulates numerous pathophysiological pathways (cell membrane signaling, neuronal polarity and inflammation) [87–89]. GSK-3β downregulates β-catenin cytosolic accumulation and then its nuclear translocation [87]. GSK-3β diminishes β-catenin, mTOR (PI3K/Akt pathway downstream), and HIF-1α expression [90].

## THE CANONICAL WNT/β-CATENIN PATHWAY IN AD

Some evidence has presented a down-regulation of the Wnt/β-catenin pathway in the pathogenesis of AD [5, 47, 91–94]. Aβ leads to a dysregulation of the WNT/β-catenin pathway in AD [95, 96]. Aβ increases Dickkopf-1 (DKK1) expression, a WNT inhibitor. In AD, DKK-1 links LRP 5/6, inhibits the complex WNT /Frd and downregulates the interaction with WNT ligands [97]. DKK-1 overexpression has been shown in AD brain of humans and transgenic mice [98]. GSK-3β activity is increased in the hippocampus of AD patients [99]. In AD, GSK-3β phosphorylates MAP tau to enhance NFTs expression [100–102]. GSK-3β over-activity is associated in AD with the diminution of β-catenin level and the increase of tau phosphorylation and NFTs formation [103]. GSK-3β activation enhances the APP cleavage [104]. The inhibition of GSK-3β activity is associated with the reversion of cell damages in AD [105].

## WNT/β-CATENIN AND GLUTAMATERGIC PATHWAY (FIGURE 2)

Some experimental studies have shown that β-catenin can regulate the expression of EAAT2, GLT-1 and GS [57, 106–108]. β-catenin knockout leads to the inhibition of glutamate neurotransmission [109]. Moreover, β-catenin expression acts in concordance with its downstream targets, as TCF/LEF, to control EAAT2 and GS expression [57]. In parallel, some studies have shown the potential role of NF-κB in the control of EAAT2 expression [110]. Evidence highlights the decrease of WNT/β-catenin pathway in rats presenting increase in neuroinflammation [91]. WNT/β-catenin pathway is mainly associated with oxidative stress and neuroinflammation [47, 111–113]. These signals, act in vicious circle with downregulated β-catenin expression, which in turn, downregulate the expression of EAAT2/GS and then, glutamate excitotoxicity [57, 114].

**Figure 2:**
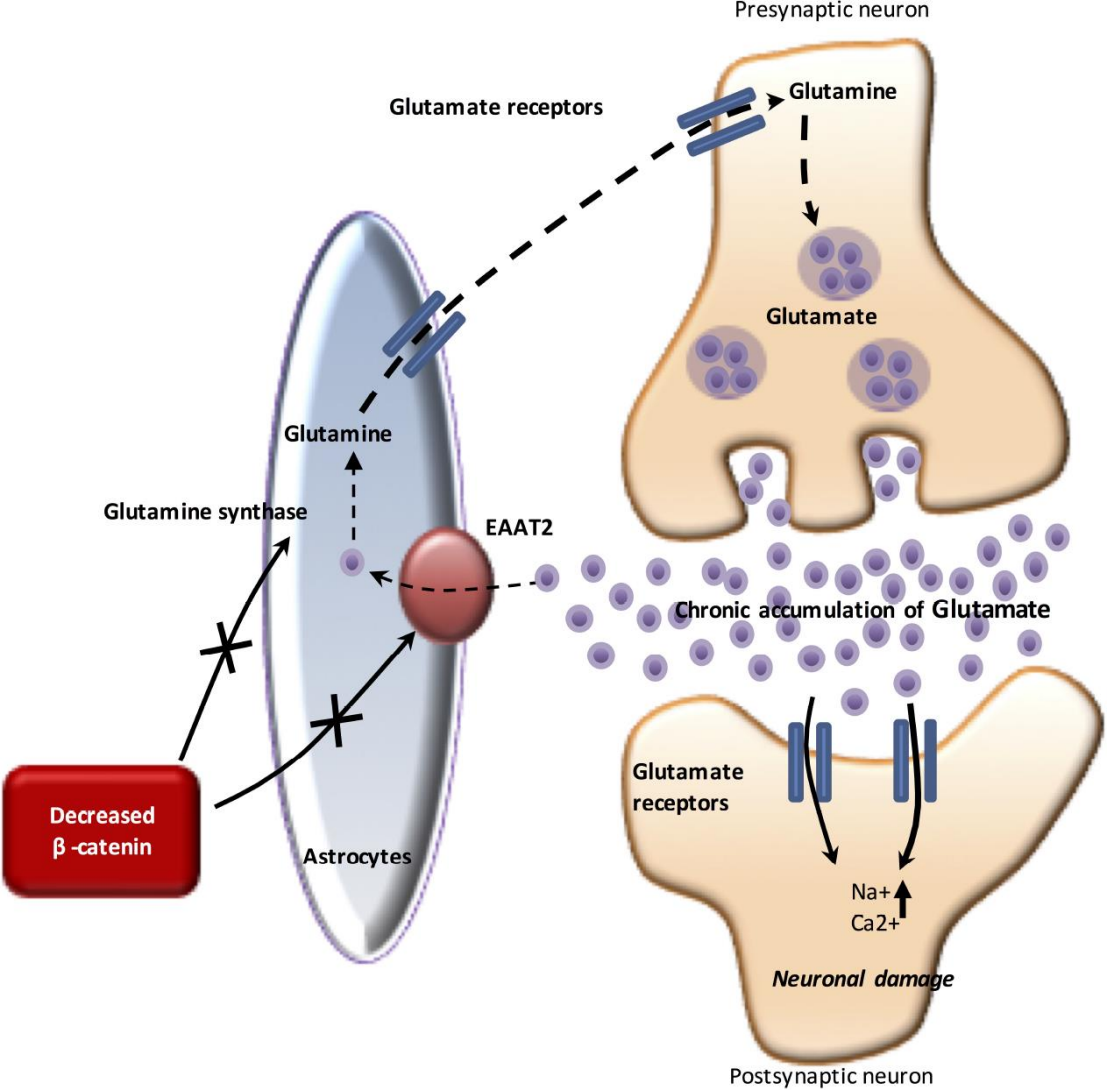
**The WNT pathway and glutamate in AD.** Under physiological conditions, glutamate released from the presynaptic neuron stimulates ionotropic glutamate receptors present on the postsynaptic neuron. The resulting influx of Na+ and Ca2+ into the cell leads to depolarization and generation of an action potential. However, chronic elevation of glutamate through impairment of EAAT2 and GS causes neuronal damage and leads to AD. In AD, the downregulation of β-catenin signaling inhibits the activity of EAAT2. Chronic accumulation of glutamate (through an impaired EAAT2 function, as glutamate reuptake function) induces excitotoxicity and then, neuronal death.

## AD: LOW ATP PRODUCTION AND DECREASED WNT/β-CATENIN PATHWAY (FIGURE 3)

Cerebral hypo-metabolism is associated with the severity of symptoms observed in AD [115]. The decrease in glucose transport in AD brains is caused by the decrease in energy demand related to the dysfunction of AD synapses [17].

**Figure 3:**
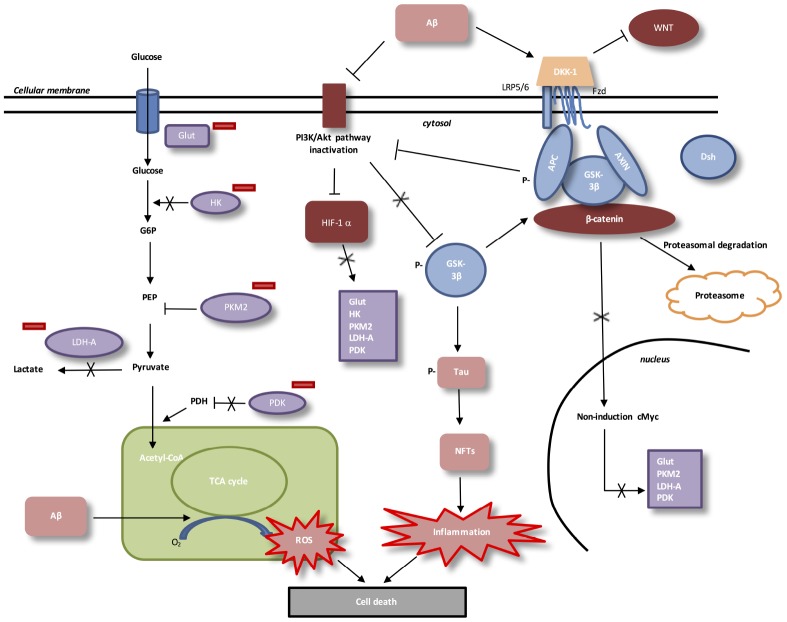
**Interactions between Aβ, WNT pathway and energy metabolism in AD.** In AD, Aβ protein activates DKK-1, an inhibitor of WNT pathway. In absence of WNT ligands, cytosolic β-catenin is phosphorylated by GSK-3β. APC and Axin combine with GSK-3β and β-catenin to enhance the destruction process in the proteasome. β-catenin does not translocate to the nucleus et does not bind TCF/LEF co-transcription factor. WNT taget genes, such as cMyc, are not activated. Aβ protein accumulation decreases level of PI3K/Akt pathway and results in inactivation of HIF-1alpha. Downregulation of beta-catenin reduces the expression of PI3K/Akt signaling. HIF-1alpha inactivated does not stimulate Glut, HK, PKM2, LDH-A and PDK1. Inactivation of HIF-1alpha involves PKM2 non-translocation to the nucleus. PKM2 inhibits PEP cascade and the formation of pyruvate. PKM2 does not bind beta-catenin and does not induce cMyc-mediated expression of glycolytic enzymes (Glut, LDH-A, PDK1). Inhibition of Glut and HK involves glucose hypo-metabolism with decreased in glucose transport and phosphorylation rates. PDK1 does not inhibit PDH, which stimulates pyruvate entrance into mitochondria. Aβ toxicity is associated with mitochondrial-derived ROS (reactive oxygen species). GSK-3β phosphorylation activates hyperphosphorylation of Tau, which induces neurofibrillary tangles and neuroinflammation.

Glut-1 (glucose transporter 1) expression, which have a main role in glucose transport in brain [116], is decreased in AD [117]. After glucose entered in cell, glucose is transformed into glucose-6-phospate by the enzyme Hexokinase (HK). Amyloidogenic AD in mouse models and in post-mortem brains show decreased levels of HK [118]. Then, glycolysis ending stage is formed by phosphoenolpyruvate (PEP) conversion into pyruvate. Tis step is catalyzed by the pyruvate kinase (PK) with an ADP. PK is composed by four isoforms (PKR, PKL, PKM1 and PKM2). Low affinity with PEP characterizes PKM2 [119].

High concentration of glucose leads to acetylation of PKM2 to reduce its activity and then, targets toward the lysosome-dependent degradation of PKM2 [120]. Peptidyl-prolyl isomerase (Pin1) allows, under high concentration of glucose, the nuclear translocation of PKM2 [120] to bind β-catenin and then, to induce c-Myc, Glut, LDH-A (lactate dehydrogenase), PDK1 (pyruvate dehydrogenase kinase 1) expression [121]. Pyruvate dehydrogenase complex (PDH) is phosphorylated by activated PDK1. Phosphorylated PDH is inactivated to prevent the conversion of pyruvate into acetyl-CoA in the mitochondria [122].

WNT/β-catenin pathway activates the PI3K/Akt pathway to increase glucose metabolism [123]. Activated PI3K/Akt pathway leads to the stimulation of hypoxia-inducible factor-1-α (HIF-1α) [124]. Thus, the overexpression of HIF-1α allows the activation of Glut, PDK1, PDH-1 and PKM2 [125–127].

In AD brain, the accumulation of Aβ is associated with the decrease of PI3K/Akt pathway [128], the decrease of WNT pathway and the degradation of β-catenin [5, 93]. In AD, β-catenin degradation leads to the reduction of PI3K/Akt pathway and then, the inactivation of HIF-1α [15, 16]. Inhibition of the activity of HIF-1alpa diminishes the nuclear translocation of PKM2 and does not allow the PEP cascade to produce pyruvate. Nuclear PKM2 does not bind β-catenin and not allows the stimulation of glycolytic enzymes. Glucose hypo-metabolism and energy deficiency is observed in AD brains [116].

## AD: ROS PRODUCTION AND DECREASED WNT/β-CATENIN PATHWAY (FIGURE 3)

PKM2 inhibition leads to increase ROS and NADPH production by inhibiting LDH-A [125]. Conversely, activation of LDH-A results in production of lactate from pyruvate [129]. This activation of LDH-A is associated with the generation of NAD^+^ to maintain NADH/ NAD^+^ redox balance [130]. A shift from mitochondrial respiration to lactate production operates and inhibits ROS production and oxidative stress [131]. Aβ toxicity is downregulated by this metabolic reprogramming with the activation of HIF-1α, PDK1 and LDH-A [132, 133]. The activation of glycolytic enzymes leads to aerobic glycolytic and then, reduces oxidative stress [133, 134].

However, Aβ toxicity is associated by the inhibition of the WNT/β-catenin pathway leading to ROS production in mitochondria [17]. FoxO (Forkhead box class O) transcription factors are main intracellular modulators of metabolic pathways including glucose transport and regulation of oxidative stress [135]. ROS decreases Wnt pathway through the diversion of β-catenin from TCF/LEF to FoxO [136]. This leads to β-catenin/FoxO complex and nuclear activation of FoxO [137, 138]. FoxO activates apoptotic genes expression [139–141] by stimulating cyclin-dependent kinase inhibitor p27, kip1 and decreasing cyclin D1 expression [142, 143]. The activation of FoxO induces apoptosis [144], whereas FoxO decreasing is associated with low Aβ exposure [145]. WNT/β-catenin pathway stimulation can phosphorylate FoxO into the cytosol and then, allows diminution of apoptosis, decrease of cytochrome c release, Bad phosphorylation and caspase signaling [146].

## AD: NEUROINFLAMMATION AND DECREASED WNT/β-CATENIN PATHWAY (FIGURE 3)

Release of cytokines, blood barrier breakdown and infiltration of leukocytes in brain characterized neuroinflammation [147]. Neurodegeneration is partly caused by the neuroinflammation [148]. NF-κB, cytokines and prostaglandins activation are responsible for CNS neuroinflammation [149, 150]. In physiologic condition, WNT/β-catenin pathway can control the immune response during neuroinflammation [151]. WNT and NF-κB act in an opposed manner [152–156]. LRP5 negatively regulates macrophage differentiation [157].

Β-catenin inhibits NF-κB-mediated transcription of pro-inflammatory genes by decreasing GSK-3β activity GSK-3β positively regulates NF-κB pathway but negatively modulates β-catenin level [158, 159]. Decreased β-catenin level is correlated with the increase of NF-κB pathway and thus, neuroinflammation [160].

## RILUZOLE AND NEURODEGENERATIVE DISEASES

Riluzole could be considered as a neuroprotective drug while its action mechanism remains unclear. Riluzole can block glutamatergic cell transmission in brain through the inhibition of the discharge of aminoakanoic acid from central nervous system. This drug can block the post synaptic effects of glutamic acid by blockage of NMDA receptors [161]. Parkinson’s disease (PD) is characterized by a mitochondrial dysfunction [94, 162, 163]. The insufficiency of energy leads to the weakness of glutamatergic activation and then contributes to PD [164]. The glutamate antagonism role of Riluzole may be useful for PD patients. Increase of synaptic efficacy of striatal ionotropic glutamatergic receptors leads to dyskinesia and may be relieved by Riluzole which acts on excitatory glutamatergic transmission [165]. Moreover, PD is associated with the decrease of the WNT/β-catenin pathway [166, 167]. Riluzole could be an interesting drug by targeting this pathway. Anxiety disorders could be reduced by anti-glutamatergic action of the Riluzole and the reduction of the amino acid neurotransmission [168]. Riluzole reduces symptoms in bipolar disorders which present a decrease in WNT/β-catenin pathway [169].

Riluzole is a well-known treatment of amyotrophic lateral sclerosis (ALS). This drug is used in ALS due to its anti-glutamatergic toxicity role while ALS presents an upregulation of the WNT/β-catenin pathway [AV].

## RILUZOLE: A POTENTIAL ACTOR ON THE DECREASED WNT/β-CATENIN PATHWAY IN AD (FIGURE 4)

Riluzole administration can counteract glutamate alterations, cognitive deficits, and tau pathology associated with P301L tau expression [24, 170]. Riluzole increases the performance in the rTg (TauP301L) 4510 mouse model of AD. The TauP301L-mediated diminution in PSD-95 expression, a compound of excitatory synapses in the hippocampus, is rescued by Riluzole. Moreover, Riluzole is an enhancer of Wnt/β-catenin pathway in both HT22 neuronal cells and adult hippocampal progenitor cells [171]. This can explain the beneficial action observed by Riluzole in AD. Riluzole has been approved for the ALS, a disease presenting an upregulation of Wnt/β-catenin pathway. the indication of Riluzole used un ALS is due to its action on the glutamatergic pathway [172]. Nevertheless, Riluzole show weak effects in median survival at 3 months [173–175]. These poor effects of Riluzole in ALS could be explained by the increasing of the WNT/β-catenin pathway by Riluzole [167]. Positive effects of Riluzole used have been observed in bipolar disorders, a disease presenting a downregulation of the WNT/β-catenin pathway [169, 176, 177]. However, only one experimental study has directly shown the positive role of Riluzole on the WNT/β-catenin pathway [171].

**Figure 4:**
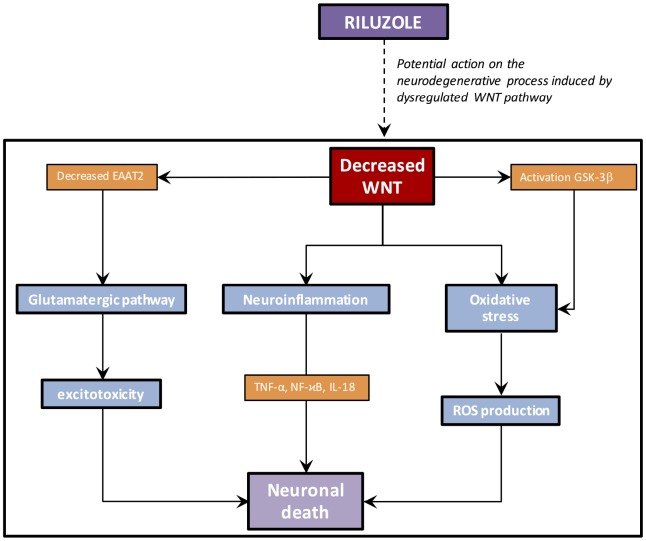
**Riluzole potential action in AD.** By directly targeting the WNT pathway, Riluzol could act on neuroinflammation, oxidative stress and the glutamatergic pathway involved in AD process.

## CONCLUSION

Primary etiology of AD remains unclear; nevertheless, neuroinflammation, oxidative stress and glutamatergic pathway could be underlying causes of AD. The canonical WNT/β-catenin pathway is downregulated in AD. The downregulation of this pathway is responsible for the enhancement of oxidative stress, neuroinflammation and the dysregulation of the glutamatergic pathway in AD. Riluzole could be an interesting therapeutic strategy in AD by targeting the WNT/β-catenin pathway and increasing it. Few studies have focused on this potential therapeutic way in AD, and futures clinical trials could highlight this interaction and the beneficial effects of Riluzole in AD.
